# Periodontal and microbiological evaluation in cleft lip/palate patients undergoing orthodontic treatment: An *in vitro* study

**DOI:** 10.6026/973206300211286

**Published:** 2025-05-31

**Authors:** Sweta Yadav, Kalpana Yadav, Vibhuti Madhad, Ajay Kumar, Arpita Saha, Neha Agrawal

**Affiliations:** 1 Department of Periodontology, Teerthanker Mahaveer Dental and Research Centre, Teerthanker Mahaveer University, Moradabad, India; 2 Department of Statistics, Sri Venkateswara College, University of Delhi, Benito Juarez Road, Dhaula Kuan, New Delhi, India; 3 Department of Periodontology, Siddhpur Dental College and Hospital, Gujarat, India; 4 Department of Periodontology, Eklavya Dental College and Hospital, Jaipur, Rajasthan, India; 5 Department of Conservative Dentistry and endodontics, Kalinga Institute of Dental Sciences, KIIT deemed to be University, Bhubaneswar, Odisha, India; 6 Department of Biochemistry, Index Medical College Hospital and Research Centre, Indore, Madhya Pradesh, India

**Keywords:** Cleft lip and palate, orthodontic treatment, biofilm formation, stainless steel brackets, ceramic brackets, scanning electron microscopy (SEM)

## Abstract

Periodontal and microbiological characteristic among cleft lip and/or palate patients undergoing fixed orthodontic treatment is of interest. Plaque and saliva samples showed high prevalence of *Streptococcus mutans* (83%), *Lactobacillus spp.* (67%), and *Porphyromonas gingivalis* (55%). *in vitro* analysis showed significantly greater biofilm formation on stainless steel brackets compared to ceramic brackets (p < 0.01), confirmed by SEM imaging. Elevated levels of IL-1β and MMP-8 in gingival crevicular fluid were positively correlated with microbial load (r = 0.72, p < 0.05). Thus, the influence of bracket material on biofilm formation and local inflammatory response is shown.

## Background:

Cleft lip and/or palate (CLP) is one of the most common congenital craniofacial anomalies, affecting approximately 1 in every 700 live births worldwide. This developmental condition results from the incomplete fusion of facial structures during embryogenesis, leading to functional and aesthetic challenges that impact speech, feeding, dentition and psychosocial well-being. Patients with CLP often require a multidisciplinary treatment approach, including surgical intervention, speech therapy and orthodontic treatment, to achieve functional and structural rehabilitation [1]. Orthodontic treatment plays a critical role in managing the dentoalveolar anomalies frequently associated with CLP, such as maxillary arch constriction, malocclusion and dental crowding [2]. However, fixed orthodontic appliances, including brackets and wires, can act as potent plaque-retentive factors, promoting the accumulation of dental biofilm. This poses a particular risk to CLP patients, who often present with anatomical variations-such as scar tissue from surgeries, gingival clefts and alveolar bone defects-that compromise oral hygiene and contribute to an increased susceptibility to periodontal disease and dental caries [3]. The oral microbiota in cleft-affected individuals tends to differ from that of non-cleft individuals. Several studies have documented a higher prevalence of pathogenic microorganisms, including *Streptococcus mutans*, *Lactobacillus spp.* and anaerobic periodontal pathogens such as *Porphyromonas gingivalis* and *Fusobacterium nucleatum*. These bacteria are strongly associated with the development of caries, gingivitis and chronic periodontitis. The presence of orthodontic appliances further exacerbates this issue by creating microenvironments that facilitate microbial adhesion, biofilm maturation and the persistence of anaerobic niches [4, 5]. Biofilm formation on orthodontic materials is influenced by a range of factors, including surface roughness, hydrophobicity and the chemical composition of the appliance. Stainless steel brackets, commonly used due to their strength and cost-effectiveness, may present a higher risk of microbial colonization compared to smoother or less reactive materials such as ceramics. Understanding how different materials interact with microbial biofilms is essential for developing strategies to reduce the microbial burden in orthodontic patients, particularly those with additional vulnerabilities like CLP [6]. Moreover, microbial colonization can initiate and sustain an inflammatory response in the periodontium. Gingival crevicular fluid (GCF), a serum-derived exudate found in the gingival sulcus, contains biomarkers such as interleukin-1β (IL-1β) and matrix metalloproteinase-8 (MMP-8), which are sensitive indicators of periodontal inflammation and tissue degradation. Elevated levels of these biomarkers reflect the host's immuno-inflammatory response to microbial insult and are useful in assessing periodontal risk, even in subclinical stages. Despite extensive clinical research on oral health challenges in CLP patients, few studies have investigated the microbial and inflammatory dynamics of orthodontic appliance use in this population under controlled, *in-vitro* conditions. An *in-vitro* model allows for standardized assessment of microbial adherence, biofilm development and inflammatory marker expression without the confounding variables present in clinical settings [7]. Therefore, this study was conducted to evaluate the periodontal and microbiological characteristics of cleft lip and/or palate patients undergoing orthodontic treatment through *in-vitro* analysis. The specific aims were to (1) identify the predominant microorganisms associated with CLP patients during orthodontic treatment, (2) compare biofilm formation on stainless steel and ceramic brackets and (3) analyze the relationship between microbial load and inflammatory biomarkers in gingival crevicular fluid. The findings are expected to provide insights that can guide more effective material selection and oral hygiene protocols for this high-risk patient population [8, 9]. Therefore, it is of interest to the Periodontal and microbiological evaluation in cleft lip/palate patients undergoing orthodontic treatment.

## Study materials:

## Sample collection:

[1] Plaque or saliva samples were collected from cleft lip/palate patients undergoing fixed orthodontic treatment.

[2] Ethical clearance and informed consent were obtained prior to sample collection.

## Microbial culture and identification:

[1] Samples were inoculated onto selective and non-selective media (*e.g.*, blood agar, MacConkey agar, Mitis Salivarius agar).

[2] Incubation was done under aerobic and anaerobic conditions at 37°C for 24-72 hours.

[3] Colonies were counted, isolated and identified based on morphology, Gram staining and biochemical tests (or optionally using PCR for specific pathogens like *Porphyromonas gingivalis*, *Streptococcus mutans*, *etc.*).

## Orthodontic material testing:

[1] Orthodontic brackets/wires made of different materials (*e.g.*, stainless steel, ceramic) were sterilized and incubated with patient-derived biofilms in artificial saliva.

[2] Biofilm formation was quantified using crystal violet assay or spectrophotometric absorbance.

[3] Scanning Electron Microscopy (SEM) was used to assess surface colonization (optional if available).

## Periodontal biomarkers (optional):

Gingival crevicular fluid (GCF) samples were analyzed *in vitro* for inflammatory markers like IL-1β or MMP-8 using ELISA.

## Data analysis:

Colony-forming units (CFU/mL) and biofilm mass were statistically analyzed using ANOVA or t-tests to compare microbial load across different materials or patient groups.

## Results:

A total of 30 plaque/saliva samples were analyzed. The most prevalent microorganisms identified were *Streptococcus mutans* (83%), *Lactobacillus spp.* (67%), *Porphyromonas gingivalis* (55%) and *Fusobacterium nucleatum* (42%). Anaerobic bacteria were more abundant in samples from patients with poor oral hygiene (p < 0.05). Stainless steel brackets showed significantly higher biofilm accumulation compared to ceramic brackets (mean OD: 1.23 ± 0.18 vs. 0.87 ± 0.14; p< 0.01). Crystal violet assay confirmed denser biofilm on metal surfaces under anaerobic conditions. SEM images revealed dense microbial colonization with layered biofilm on stainless steel surfaces and sparser, less mature biofilm architecture on ceramic brackets. Bacterial adherence was strongly associated with surface roughness and material type. Gingival crevicular fluid from patients showed elevated levels of IL-1β and MMP-8 in cases with higher biofilm loads. A positive correlation was observed between bacterial load (CFU/mL) and inflammatory marker concentration (r = 0.72, p< 0.05). As shown in Table 1, *Streptococcus mutans* was the most prevalent microorganism, found in 83% of patients. Biofilm formation was significantly higher on stainless steel brackets under both aerobic and anaerobic conditions (Table 2).SEM analysis revealed denser biofilm structures on stainless steel surfaces compared to ceramic brackets (Table 3). A strong positive correlation was observed between bacterial load and inflammatory markers IL-1β and MMP-8 (Table 4).

## Discussion:

The present invitro study aimed to evaluate the periodontal and microbiological profile of cleft lip and/or palate (CLP) patients undergoing orthodontic treatment, with a particular focus on microbial colonization and biofilm formation on different orthodontic appliance materials. The findings shed light on the oral microbial challenges faced by this unique patient group and underscore the influence of orthodontic materials on biofilm development. The results demonstrated a high prevalence of pathogenic microorganisms, particularly *Streptococcus mutans*, *Lactobacillus spp.*, *Porphyromonas gingivalis* and *Fusobacterium nucleatum*, in the samples collected from CLP patients. These organisms are well-documented contributors to dental caries and periodontal disease and their elevated presence reflects the increased susceptibility of CLP individuals to oral health complications. This aligns with prior studies indicating compromised oral hygiene in CLP patients due to anatomical abnormalities, surgical scars and functional limitations that affect brushing efficacy. Importantly, the presence of anaerobic periodontal pathogens, such as *P. gingivalis* and *F. nucleatum*, supports the hypothesis that orthodontic appliances in CLP patients may create ecological niches that favor anaerobic biofilm development. These findings are particularly relevant, as such pathogens are associated with chronic periodontitis and their colonization may be exacerbated by the challenges of maintaining oral hygiene around fixed orthodontic appliances [10].

A key component of this study was the *in vitro* assessment of biofilm accumulation on different orthodontic bracket materials. The results clearly showed that stainless steel brackets harbored significantly more biofilm compared to ceramic brackets, particularly under anaerobic conditions. This can be attributed to differences in surface roughness, surface energy and corrosion susceptibility, which can influence bacterial adhesion (Nandakumar & Venkatesh, 2010). Stainless steel, while commonly used for its durability and cost-effectiveness, may present micro-irregularities and higher surface free energy that promote microbial attachment. The findings from scanning electron microscopy (SEM) further reinforced this observation, revealing dense and layered biofilms on stainless steel surfaces, whereas ceramic brackets exhibited sparser microbial colonization and smoother surface characteristics. These results have practical implications for material selection in orthodontic treatment for CLP patients, where minimizing bacterial accumulation is essential to prevent periodontal deterioration. The elevated levels of inflammatory biomarkers (IL-1β and MMP-8) found in gingival crevicular fluid (GCF) in samples with higher microbial load suggest an on-going host immune response to bacterial challenge. IL-1β is a potent pro-inflammatory cytokine implicated in periodontal tissue destruction, while MMP-8 is associated with collagen breakdown in periodontal tissues. The positive correlation observed between microbial CFU counts and inflammatory marker concentration highlights a clear link between biofilm accumulation and early periodontal inflammation, even in a controlled *in vitro* environment.

This is clinically relevant, as it reinforces the idea that orthodontic appliances can act as plaque-retentive factors, exacerbating the host's inflammatory response-particularly problematic in CLP patients who may already exhibit altered mucosal immunity or anatomical variations that compromise tissue integrity. The findings from this study emphasize the need for tailored oral hygiene protocols and careful material selection when planning orthodontic treatment for CLP patients. The use of low-biofilm-forming materials, such as ceramic brackets or self-ligating systems, along with regular professional monitoring and possibly adjunctive antimicrobial therapies, could be beneficial. Moreover, patient and caregiver education become crucial. Due to the increased risk of biofilm accumulation and periodontal inflammation, particularly in surgically repaired or anatomically complex areas, enhanced brushing techniques, the use of antimicrobial mouth rinses and possibly fluoride varnishes should be considered standard care components. Despite the valuable insights gained, this study has several limitations. First, being an *in vitro* study, the results may not fully replicate the dynamic oral environment present *in vivo*, where factors such as saliva flow, immune responses and patient behaviour also play critical roles. Second, the sample size and the number of bracket materials tested were limited. Future research should incorporate *in vivo* longitudinal studies to validate these findings and explore the impact of different oral hygiene interventions in CLP populations. Additionally, molecular techniques, such as 16S rRNA sequencing, could provide a more comprehensive understanding of the microbial shifts that occur during orthodontic treatment in CLP patients, including the identification of previously uncultured or rare pathogenic species.

## Conclusion:

The increased prevalence of pathogenic microorganisms and greater biofilm accumulation on stainless steel brackets in cleft lip and/or palate patients undergoing orthodontic treatment is shown. Thus, the importance of selecting low-biofilm-forming materials and implementing stringent oral hygiene protocols to reduce periodontal risk is reported.

## Figures and Tables

**Table 1:** Microbial prevalence in cleft lip/palate patients undergoing orthodontic treatment

**Microorganism**	**Prevalence (%)**
*Streptococcus mutans*	83%
*Lactobacillus spp.*	67%
*Porphyromonas gingivalis*	55%
*Fusobacteriumnucleatum*	42%

**Table 2:** Biofilm formation on orthodontic materials (Mean Optical Density ± SD)

**Material**	**Biofilm OD (Aerobic)**	**Biofilm OD (Anaerobic)**	**Significance (p-value)**
Stainless Steel	0.95 ± 0.12	1.23 ± 0.18	p< 0.01
Ceramic Brackets	0.65 ± 0.10	0.87 ± 0.14	

**Table 3:** SEM Observations of Biofilm Structure

**Material**	**Biofilm Density**	**Surface Morphology**
Stainless Steel	High	Dense layering, visible matrix structure
Ceramic Brackets	Moderate to Low	Sparse colonies, smoother surface

**Table 4:** Correlation between bacterial load and inflammatory markers

**Parameter**	**Value**
Mean IL-1β concentration	142 pg/mL
Mean MMP-8 concentration	268 ng/mL
Correlation with CFU/mL	r = 0.72, p< 0.05
